# Inhibition of the platelet-derived growth factor receptor beta (PDGFRB) using gene silencing, crenolanib besylate, or imatinib mesylate hampers the malignant phenotype of mesothelioma cell lines

**DOI:** 10.18632/genesandcancer.129

**Published:** 2017-01

**Authors:** Ombretta Melaiu, Calogerina Catalano, Chiara De Santi, Monica Cipollini, Gisella Figlioli, Lucia Pellè, Elisa Barone, Monica Evangelista, Alice Guazzelli, Laura Boldrini, Elisa Sensi, Alessandra Bonotti, Rudy Foddis, Alfonso Cristaudo, Luciano Mutti, Gabriella Fontanini, Federica Gemignani, Stefano Landi

**Affiliations:** ^1^ Paediatric Haematology/Oncology Department, IRCCS, Ospedale Pediatrico Bambino Gesù, Rome, Italy; ^2^ Department of Biology, University of Pisa, Pisa, Italy; ^3^ Division of molecular genetic epidemiology, German Cancer Research Center (DKFZ), Heidelberg, Germany; ^4^ Department of Medicine, Education and Research Centre, Royal College of Surgeons in Ireland, Beaumont Hospital, Dublin, Ireland; ^5^ IFOM, the FIRC Institute of Molecular Oncology, Milan, Italy; ^6^ Institute of Clinical Physiology (IFC), CNR, Pisa, Italy; ^7^ School of Environment and Life Sciences, University of Salford, Manchester, United Kingdom; ^8^ Department of Surgical, Medical, Molecular Pathology and Critical Care, Division of Pathological Anatomy, University of Pisa, Pisa, Italy; ^9^ Preventive and Occupational Medicine, University Hospital of Pisa, Pisa, Italy; ^10^ Department of Translational Research and of new Technologies in Medicine and Surgery, University of Pisa, Pisa, Italy

**Keywords:** Malignant Pleural Mesothelioma, therapeutic targets, PDGFRB, RNA interference, drug inhibitors

## Abstract

Malignant pleural mesothelioma (MPM) is a cancer of the pleural cavity resistant to chemotherapy. The identification of novel therapeutic targets is needed to improve its poor prognosis. Following a review of literature and a screening of specimens we found that platelet-derived growth factor receptor beta (*PDGFRB*) is over-expressed, but not somatically mutated, in MPM tissues. We aimed to ascertain whether *PDGFRB* is a MPM-cancer driver gene. The approaches employed included the use of gene silencing and the administration of small molecules, such as crenolanib and imatinib (PDGFR inhibitors) on MPM cell lines (IstMes2, Mero-14, Mero-25). Met5A cells were used as non-malignant mesothelial cell line. *PDGFRB*-silencing caused a decrease in the proliferation rate, and a reduced colony formation capacity, as well as an increase of the share of cells in sub-G_1_ and in G_2_ phase, and increased apoptotic rate of MPM cell lines. Loss of migration ability was also observed. Similar, or even further enhanced, results were obtained with crenolanib. Imatinib showed the least effective activity on the phenotype. In conclusion, our study highlights PDGFRB as target with a clear role in MPM tumorigenesis and provided a rationale to explore further the efficacy of crenolanib in MPM patients, with promising results.

## INTRODUCTION

Malignant pleural mesothelioma (MPM) is a cancer of the pleural cavity with a poor prognosis and the identification of novel therapeutic targets is urgently needed. Recently, our research group investigated the expression status of 119 candidate cancer genes in MPM tissues and cell lines. Among the identified genes, one of the most interesting was *PDGFRB* encoding for the platelet-derived growth factor receptor beta, already suggested as a MPM-cancer gene by previous research groups [1,2]. PDGF is composed of homo-dimers or hetero-dimers of two polypeptide chains, denoted A and B. Two different PDGF receptors, alpha and beta, have been described [3,4]. The two receptor subtypes show different affinities for the dimeric PDGF isoforms. The PDGF-alpha receptor binds with high affinity all three forms (i.e. AA, AB, and BB), whereas the beta-receptor subtype only binds PDGF-BB [5,6]. It is a confirmed observation that panels of MPM cell lines express preferentially PDGF beta-chain and PDGF beta-receptor transcripts, whereas normal mesothelial cell lines do not express PDGF B-chain mRNA and little or no PDGF beta-receptor mRNA [7]. In contrast, normal mesothelial cell lines were found to express PDGF alpha-receptor mRNA, which could not be detected in mesothelioma cell lines [7]. It has been suggested that the PDGF/PDGFR-beta interaction could be involved in the carcinogenesis of various tissues, including osteosarcoma [8], meningiomas, melanomas, neuroendocrine tumors, ovarian, pancreatic, gastric, lung, prostate cancers [9], and MPM [10], with both autocrine and paracrine mechanisms of growth stimulation. In agreement with this, it has been shown that PDGFRB is also associated with the aggressive behavior of several types of tumors. The 60% of colon cancer patients express high levels of this gene and the PDGFRB expression correlates with lymphatic dissemination of this cancer [11]. Steller EJ showed that PDGFRB signaling in mesenchymal-like tumor cells (as colorectal cancer cells) contributes to invasion and liver metastasis formation [12]. High PDGFRs expression correlates with advanced stage disease and poor prognosis in breast [13], liver [14], and pancreatic carcinomas [15].

Given the role of PDGFRB in cancer, a plethora of PDGF/PDGFR pathway inhibitors are available and assayed in clinical trials for leukemia, gastrointestinal stromal tumors (GIST), and glioma (https://clinicaltrials.gov/). Thus, it is of interest to explore whether MPM patients may also benefit from the use of these agents. To this end, in the present work we studied the effect of *PDGFRB* inhibition in MPM cell lines. The approaches included the use of gene silencing and PDGFRB inhibitors. The results support PDGFRB up-regulation as a cancer-driver mechanism and suggest this receptor as a candidate therapeutic target worth to be exploited in the treatment of this disease.

## RESULTS

### PDGFRB somatic mutation screening

Given that previous works and our investigations highlighted that between 20-40% of MPM specimens over-express PDGFRB [2,16,17], we wondered whether MPM tissue samples may bear somatic mutations within the *PDGFRB* locus. Thus, the tyrosine kinase loop domain encoded by exons 12-18 was screened on a series of 96 MPM specimens. We found only a common polymorphism, but no somatic mutations (Table S1). Next, to further study the possible mechanism of PDGFRB over-expression in MPM, we analyzed the copy number alterations of the *PDGFRB* genomic region in 83 MPM patients whose data were deposited in The Genome Cancer Atlas database (TGCA, URL at http://cancergenome.nih.gov/cancersselected/Mesothelioma). Even in this case, any significant amplification was detected in correspondence of *PDGFRB* genomic region (data not shown for brevity).

### PDGFRB expression in MPM cell lines

**Figure 1:**
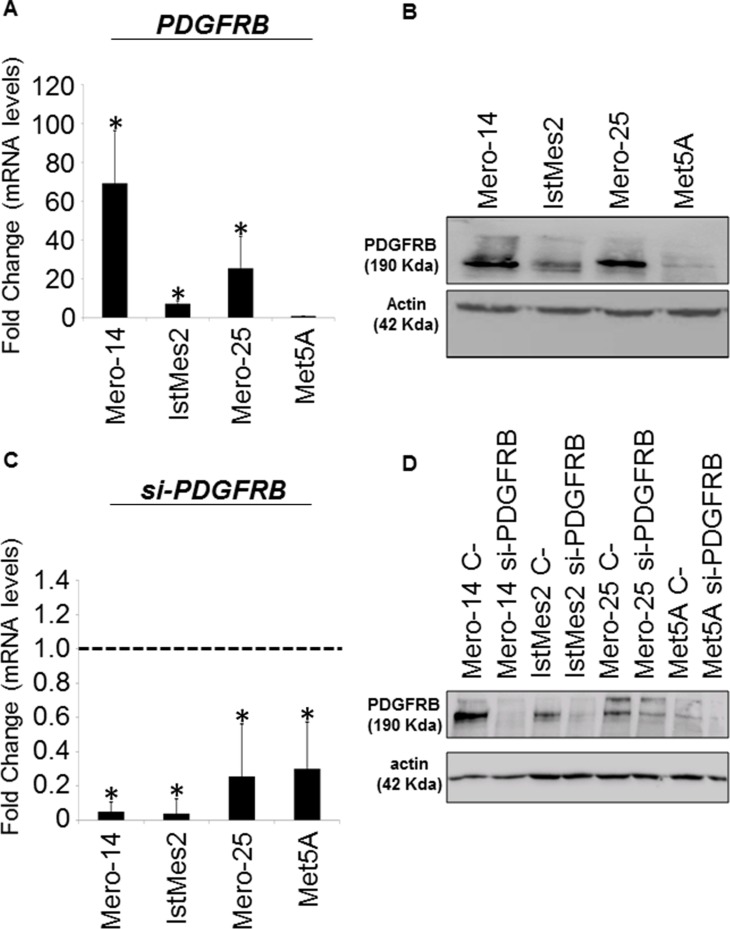
Expression levels of *PDGFRB* in Mero-14, IstMes2, and Mero-25 human MPM cell lines and Met5A A. RT-qPCR showing the mRNA expression levels of *PDGFRB* measured on MPM cell lines and related to Met5A cells (set to 1). *RPLP0*, *HPRT*, and *TBP* were used for normalization. Error bars show the standard error of the mean (SEM) from three independent experiments, each performed in triplicate. B. Protein levels of PDGFRB in Mero-14, IstMes2, Mero-25, and Met5A cells. β-actin was used as reference. The protein levels were confirmed by two independent experiments. C. RT-qPCR showing the mRNA expression levels of *PDGFRB* in Mero-14, IstMes2, Mero-25, and Met5A cells, after siPDGFRB administration, related to their own C-PDGFRB (set to 1, dotted black bar). *RPLP0*, *HPRT*, and *TBP* were used for normalization. Errors bars are SEM, from three independent experiments, each performed in triplicate. D. Protein levels of PDGFRB after its depletion in Mero-14, IstMes2, Mero-25, and Met5A cells. β-actin was used as reference. The protein levels were confirmed by two independent experiments.

The expression level of *PDGFRB* was screened on a panel of three human MPM cell lines: Mero-14, Mero-25, and IstMes2. The SV40-immortalized Met5A cell line was used as a model of non-malignant mesothelial cells. As shown in Figure 1A, all MPM cells showed up-regulated PDGFRB expression. Mero-14 cells showed the highest amount of mRNA expression level of around 70-fold compared to that of the Met5A cell line. Mero-25 cells showed an increased expression of about 30-fold, whereas IstMes2 cells had a 10-fold increase. All these differences were statistically significant using Met5A as reference (P=6×10^−3^, P=0.01, and P=3×10^−3^, respectively). Protein expression analysis gave similar results. The highest levels of PDGFRB protein was found in Mero-14 cells with an average increase of 99% when compared to Met5A cells. Mero-25 cells showed an increase of 97%, whereas the IstMes2 cell line had an increase of 70%, as shown in Figure 1B. To study further the role of PDGFRB gene, all four cell lines underwent RNA interference (RNAi). The silencing efficiency was measured at mRNA and protein levels. Mero-14 and IstMes2 cells showed a reduction of PDGFRB expression of about 95% whereas the Mero-25 cell line showed a silencing efficacy of about 50%. Met5A cells showed at least 70% PDGFRB depletion at the protein level, although the quantification was difficult given the minimal PDGFRB expression before RNAi. The results are shown both at mRNA and protein levels in Figure 1C and D, respectively.

### PDGFRB silencing and cellular growth

**Figure 2:**
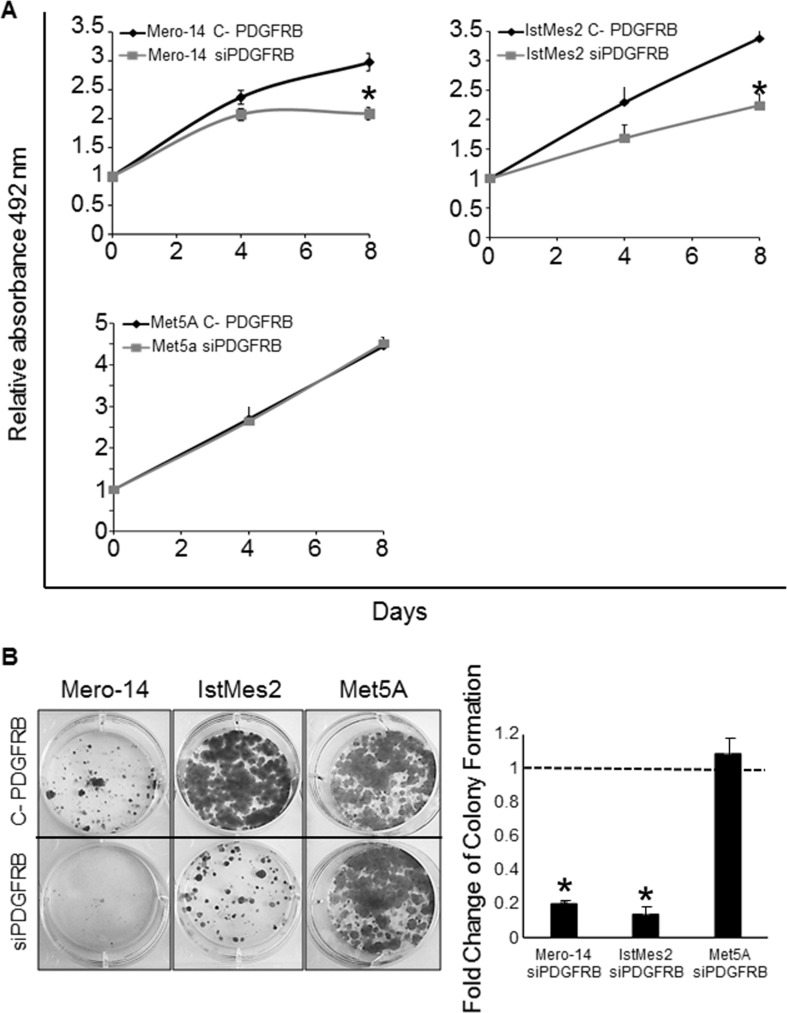
Role of PDGFRB in cellular growth A. SRB proliferation assay in Mero-14, IstMes2 and Met5A cells treated with 50 nM of the C-PDGFRB or siPDGFRB. Error bars represent SEM of three independent experiments, each performed in quadruplicate. B. Clonogenic assay: representative micrographs of colonies formed by C-PDGRB (top) and siPDGFRB (bottom) transfected Mero-14, IstMes2 and Met5A cell lines on the left and the corresponding histograms of each measurement on the right. Two different experiments were performed, each in triplicate.

SRB assay was performed to evaluate the effect of PDGFRB silencing on cellular growth of MPM cell lines. Following the administration of siPDGFRB, the proliferation rate was significantly reduced for Mero-14 (P=2×10^−3^) and IstMeS2 (P=6.7×10^−3^) cells, when compared to cells treated with C-PDGFRB. This reduction was particularly evident from the third day of treatment, and further decreased on day 8 by 30% and 34% respectively (Figure 2A). Mero-25 did not show any reduction, whereas the depletion of *PDGFRB* did not produce any effect on Met5A cells. In parallel with the measurement of the proliferative capabilities of cells, a colony formation assay was used to evaluate the clonogenic capacities of silenced cells. As shown in Figure 2B, *PDGFRB* silencing had a strong effect on abolishing clonogenicity in Mero-14 (−80.1%; P=2.9×10^−3^) and IstMes2 (-86.3%, P=5×10^−4^), when compared to control C-PDGFRB-transfected cells. A slight reduction of colony formation capacity was also observed in Mero-25 cells after PDGFRB silencing, but this decrease was not statistically significant (data not shown). No effect was detected on the non-MPM cells Met5A.

### PDGFRB silencing and cell cycle progression

The effect of *PDGFRB*-silencing on the progression of cell cycle was evaluated through flow cytometry analysis. In IstMes2 cell line, siPDGFRB induced an increase of the share of cells in sub-G_1_ (P=0.03), a decrease of cells in G_0_/G_1_ phase (P=1.4×10^−3^), and an increase of the share of cells in G_2_/M checkpoint phase (P=10^−3^), as compared to the same cells treated with C-PDGFRB. A similar trend, not statistically significant, was also shown for Mero-14 cells. No effects were detected on Met5A and Mero-25 cells (Table S2, A).

### PDGFRB silencing and apoptosis

**Figure 3:**
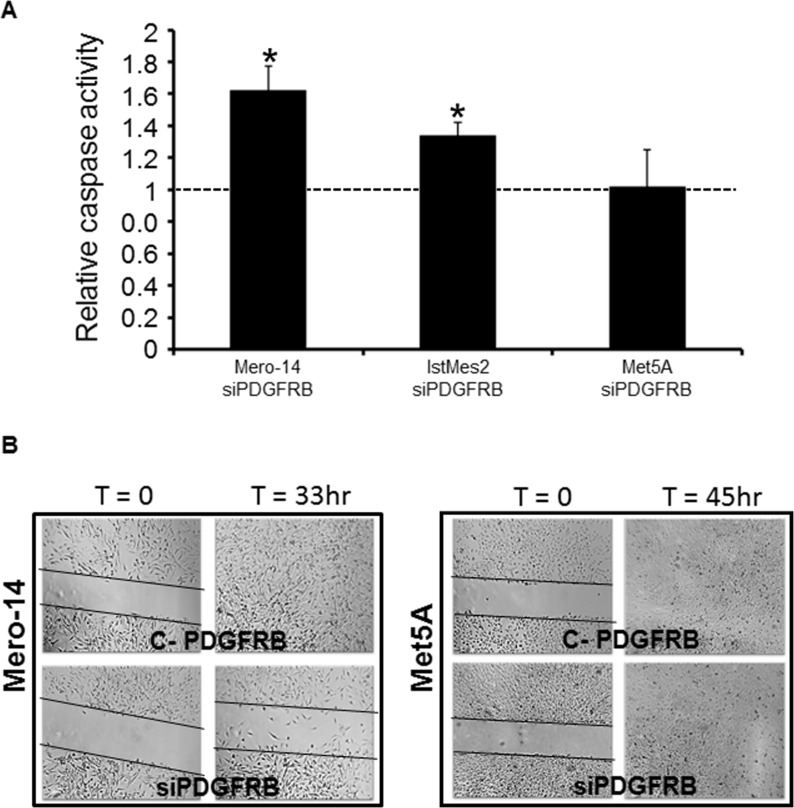
**A.** Role of PDGFRB in apoptosis. Caspase activity measured on Mero-14, IstMes2, and Met5A cells transfected with 50 nM of C-PDGFRB (set to 1, black bar), or siPDGFRB. A marked increase in apoptosis is observed for Mero-14, IstMes2 cells, but not for the non-MPM Met5A cells. Error bars represent SEM of three independent experiments, each performed in triplicate. B. Role of PDGFRB on cellular migration. Confluent monolayers of Mero-14 (left) and Met5A (right) cells transfected with 50 nM of C-PDGFRB, or siPDFRB, respectively. Two different experiments were carried out, each performed in triplicate. Massive effect was observed in the wound-healing assay, following siRNA transfections, for Mero-14 cells. Met5A did not respond to siPDGFRB in the migration ability.

The activity of caspases 3 and 7 was measured for the evaluation of the apoptotic rate following PDGFRB silencing. After the transfection with siPDGFRB, Mero-14 and IstMes2 cell lines showed an average increase of caspase activity of 62% and 34%, respectively (C-PDGFRB used as control P<10^−3^ and P=9×10^−3^, respectively). The increase of apoptotic cells in IstMes2 was of the same order of magnitude as the increase of sub-G_1_ cells measured with flow cytometry. No changes were observed in Mero-25 and MeT5A cells (Figure 3A).

### PDGFRB silencing and migration capacity

Wound-healing assay was employed to assess the effect of PDGFRB silencing on cellular migration of MPM cell lines. No statistically significant differences in migration were observed in Mero-25, IstMeS2, and Met5A cells following the siPDGFRB administration. Nevertheless, Mero-14 cells showed a statistically significant reduced migration ability, as compared to C-PDGFRB, at 72 h after PDGFRB depletion (P=0,019) (Figure 3B).

### PDGFRB silencing and anchorage-independent growth capacity

**Figure 4:**
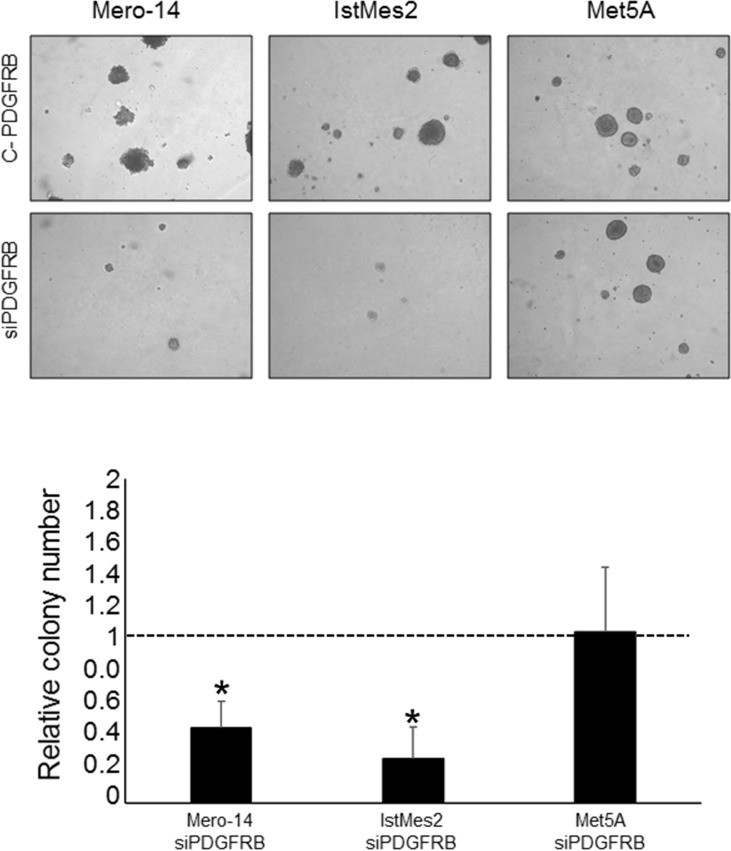
Role of PDGFRB in anchorage-independent growth capacity following siPDGFRB transfection Representative images of soft agar colonies formed by C-PDGRB and siPDGFRB transfected Mero-14, IstMes2 and Met5A cell lines (top) and the corresponding histograms of each measurement (bottom). Error bars represent SEM of two independent experiments, each performed in triplicate.

Soft agar colony formation assay was performed to evaluate the malignant transformation potency on anchorage-independent growth of MPM cells with or without the administration of siPDGFRB. Colony formation on soft agar was observed in C-PDGFRB cells (Figure 4), whereas siPDGFRB treatment significantly decreased both size and number of soft agar colonies formed by Mero-14 and IstMes2 cells (P=0.006 and P=0.007 respectively). Mero-25 cells were not able to form colonies on soft agar in both treated and control conditions, even after 28 days of culture (data not shown). No significant differences were detected in Met5A cell line. Hence the results of the soft agar assay confirmed that PDGFRB depletion decreased *in vitro* tumorigenic potential of MPM cells.

### PDGFRB drug inhibitors and cellular growth

**Figure 5:**
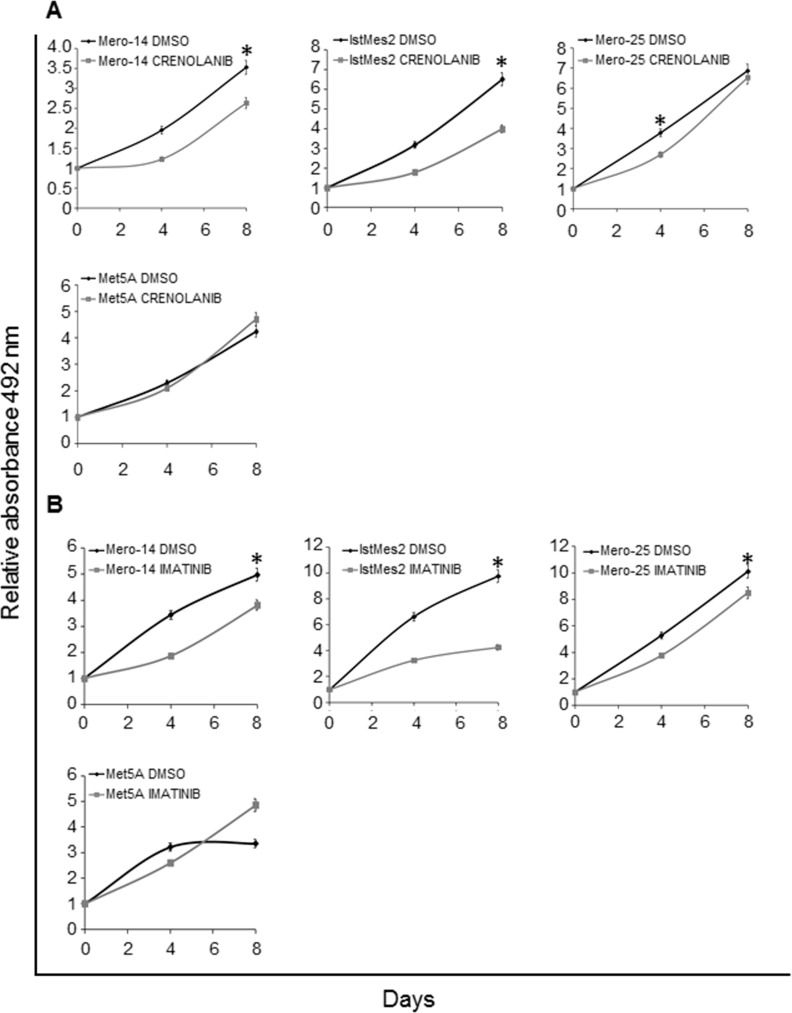
Role of PDGFRB in cellular growth, following treatment with chemotherapeutic drugs A. Proliferation assay in Mero-14, IstMes2, Mero-25, and Met5A cells treated with DMSO or 1μM of crenolanib. Error bars represent SEM of three independent experiments, each performed in quadruplicate. B. Proliferation assay in Mero-14, IstMes2, Mero-25, and Met5A cells treated with DMSO or 25μM of imatinib. Error bars represent SEM of three independent experiments, each performed in quadruplicate.

We next tested two PDGFRB inhibitors: crenolanib and imatinib. These small molecules are known to act by preventing PDGF-induced PDGFR autophosphorylation, and their effect on MPM cell lines was then compared. Similar to that found after *PDGFRB* gene-silencing, reductions of proliferative capabilities were observed, where the strongest activity of crenolanib was found on Mero-14 and IstMes2 cells, as reported in Figure 5A. The proliferation rate of Mero-14 cells suffered a reduction of about 25% (statistically significant, P=0.03), whereas IstMes2 cells reached a 40% reduction (P=0.04). Mero-25 cells seemed poorly sensitive to crenolanib, as the growth rate was only reduced slightly. No effect was found on Met5A cells. In this respect, the response of all cell lines was overlapping to that observed after siPDGFRB administration. Similar results were obtained after treatment with imatinib (Figure 5B), but with 25-fold higher doses. When an arbitrarily chosen and intermediate concentration of 8 μM for both molecules was employed, as expected, the inhibitory effect of crenolanib was much stronger, whereas imatinib was almost ineffective (P = 10^−4^, data not shown).

**Figure 6:**
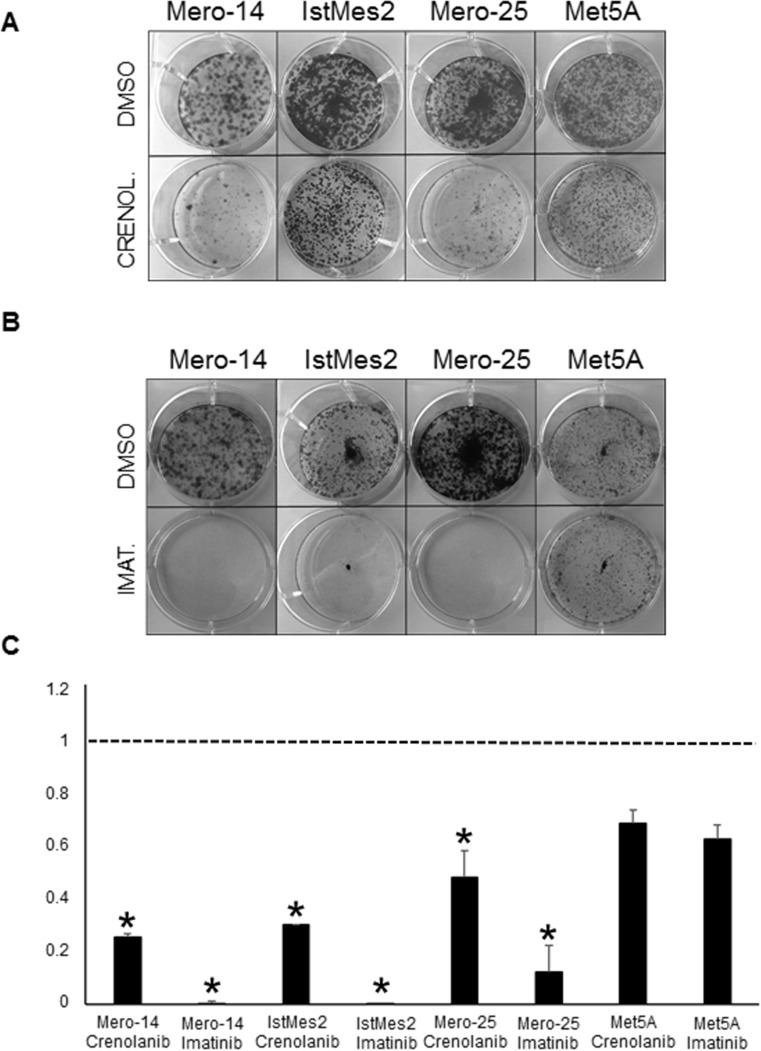
Role of PDGFRB in cellular growth, following treatment with crenolanib, and imatinib A. Clonogenic assay: representative micrographs of colonies formed by Mero-14, IstMes2, Mero-25, and Met5A cell lines treated with DMSO (top) and crenolanib (1μM, bottom). B. Clonogenic assay: representative micrographs of colonies formed by Mero-14, IstMes2, Mero-25, and Met5A cell lines treated with DMSO (top) and imatinib (25μM, bottom). C. Histograms corresponding to each measurement performed for crenolanib and imatinib. Two different experiments were performed, each in triplicate.

Crenolanib (at 1μM) and imatinib (at 25μM) were both efficient in abolishing the colony formation capacity of Mero-14 (P_crenolanib_ = 2×10^−3^; P_imatinib_ = 6.3×10^−10^), IstMes2 (P_crenolanib_ = 2.3×10^−3^; P_imatinib_ =1.48×10^−8^), and Mero-25 cells (P_crenolanib_=0.02; P_imatinib_=2×10^−4^), without any statistically significant effect on the clonogenic survival of the Met5A cell line (Figure 6).

### PDGFRB drug inhibitors and cell cycle progression

Similar to the observations made following treatment with siPDGFRB, the flow cytometry analysis showed that crenolanib induces an increase in the share of cells in sub-G_1_ for Mero-14 (P=2×10^−3^) and IstMes2 (P=0.04), a reduced share of cells in G_0_/G_1_ phase (P=1×10^−3^ for Mero-14 cells, and P=0.05 for IstMes2 cells), and an accumulation in G_2_/M phase for IstMes2 cells (P=0.04). In addition, an accumulation in G_2_/M phase was also observed for Mero-25 cells (P=0.01). The treatment with imatinib affected the cell cycle progression with lower efficacy compared to crenolanib: a statistically significant accumulation (P=0.02) of cells in G_2_/M phase was found only for IstMes2 cell line. Met5A did not experience significant changes in the cell cycle progression during the treatment with either of these drugs (Table S2, B-C).

### PDGFRB drug inhibitors and apoptosis

**Figure 7:**
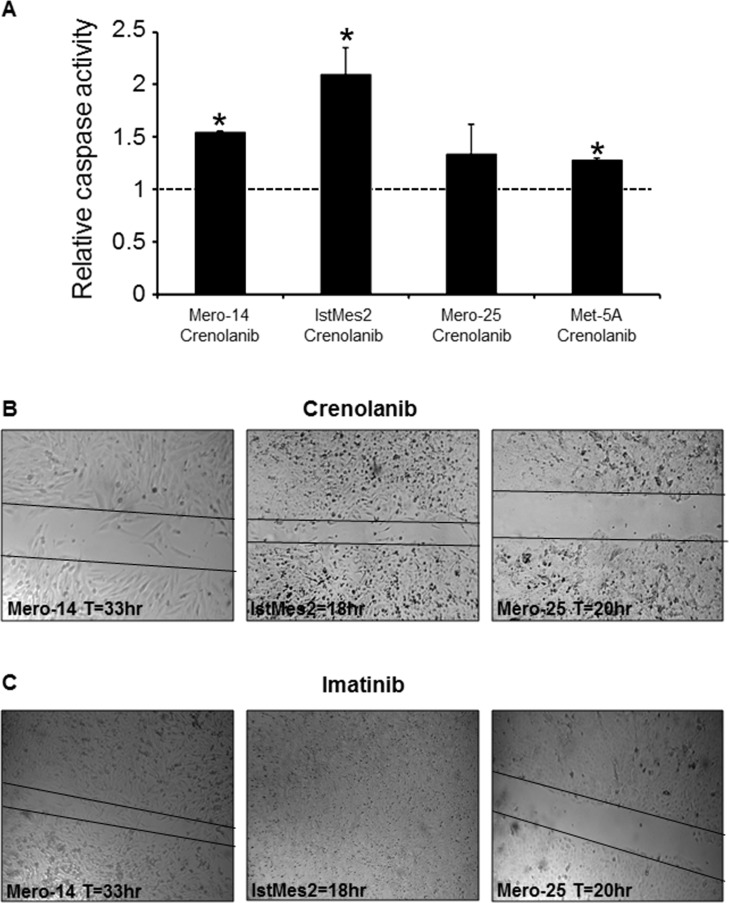
**A.** Role of PDGFRB in apoptosis, following treatment with crenolanib. Caspase activity measured on Mero-14, IstMes2, Mero-25, and Met5A cells treated with DMSO (set to 1, dotted black bar), or crenolanib (1μM). A marked increase in apoptosis is observed for Mero-14 and IstMes2 cells. A slight induction of apoptosis is also reach by Mero-25 and Met5A cells, after treatment with crenolanib. Error bars represent SEM of three independent experiments, each performed in triplicate. B. Role of PDGFRB in cellular migration, following treatment with crenolanib, and imatinib. Confluent monolayers of Mero-14, IstMes2, and Mero-25 cells treated with 1μM of crenolanib (top), or 25μM of imatinib (bottom). Massive effect was observed in the wound-healing assay, following crenolanib treatment for Mero-14, IstMes2, and Mero-25 cells. Imatinib treatment impaired the migration ability of Mero-14 and Mero-25 cells, but not of IstMes2 cells. Two independent experiments were carried out, each performed in triplicate.

The caspase 3 and 7 activities were assayed on the four cell lines, after 72 h of a crenolanib or imatinib continuous treatment. With crenolanib at 1μM (Figure 7A), Mero-14 and IstMes2 showed a strong and statistically significant increase of apoptosis (+54%, P=3.8×10^−3^, and +100% P=0.017, respectively), in agreement with the share of sub-G_1_ cells observed with flow cytometry analysis. Mero-25 showed an increased apoptosis rate (+34%), but this was not statistically significant (P=0.12). Met5A cells had increased apoptosis (+20%), slightly statistically significant, and not confirmed with the share of sub-G_1_ cells observed with flow cytometry analysis. Imatinib treatment was not associated with increased apoptosis in any treated cell line.

### PDGFRB drug inhibitors and migration

The cell migration ability of MPM cells was heavily compromised by treatment with crenolanib. Mero-14, IstMes2, and also Mero-25 treated cells showed a statistically significant difference in the closure of the scratch compared to the corresponding controls (Figure 7B; P=0.04; 0.02; 7×10^−3^ respectively). Imatinib treatment affected the migration capacity of Mero-25 cells with statistical significance (P=4×10^−3^), but not that of Mero-14 and IstMes2 cells (Figure 7C), compared with their corresponding controls. Met5A cells were not able to close the scratch completely, and the difference with their corresponding control was not statistically significant.

### PDGFRB drug inhibitors and anchorage-independent growth capacity

**Figure 8:**
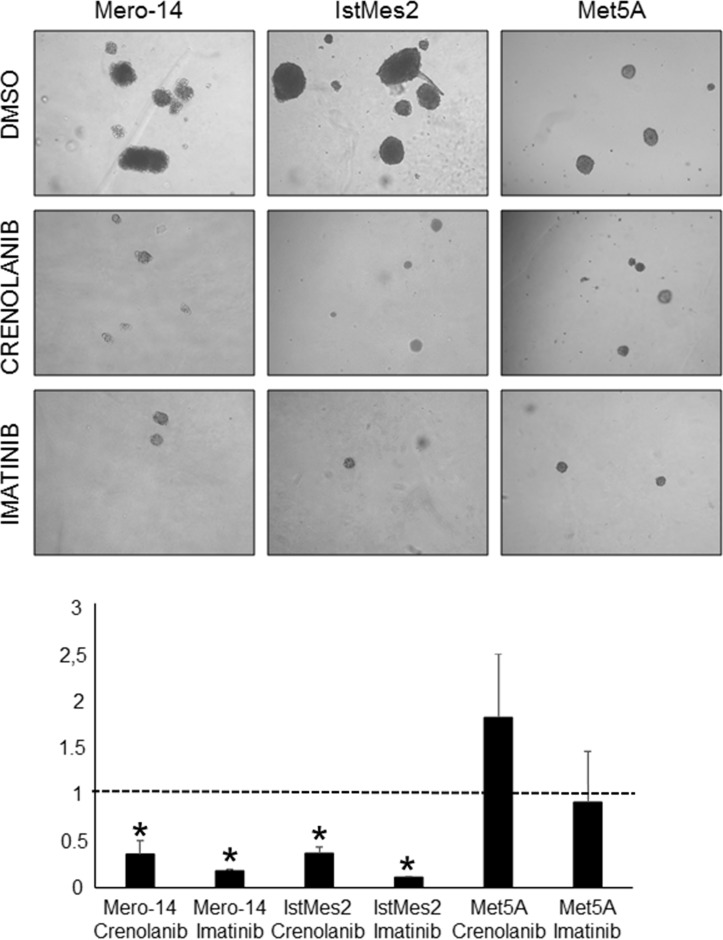
Role of PDGFRB in anchorage-independent growth capacity following drugs inhibitors treatments Representative images of soft agar colonies formed by DMSO, crenolanib and imatinib treated Mero-14, IstMes2 and Met5A cell lines (top) and the corresponding histograms of each measurement (bottom). Error bars represent SEM of two independent experiments, each performed in triplicate.

Crenolanib and imatinib treatment significantly decreased both size and number of soft agar colonies formed by Mero-14 (P_crenolanib_ = 6×10^−5^; P_imatinib_ = 4×10^−7^) and IstMes2 cells (P_crenolanib_ = 0.02; P_imatinib_ = 0.007) similar to that observed for siPDGFRB treatments. Mero-25 cells did not form colonies in any of the treatment conditions, nor in control (data not shown). Met5A did not show differences in soft agar colony formation capacity when treated with crenolanib compared to DMSO. These cells were more affected by imatinib treatment, forming smaller colonies than those of the control. However, the difference is not statistically significant (Figure 8).

## DISCUSSION

In this work we studied somatic mutations within *PDGFRB* and the inhibition of PDGFRB on MPM. No activating gene mutations were detected and these results are in agreement with that found both in COSMIC (http://cancer.sanger.ac.uk/cosmic) and TGCA databases. The former reports that, unlike *PDGFRA*, *PDGFRB* gene is not frequently mutated at somatic level in cancer tissues (only 276 simple mutations in a total of 26.218 tested samples), the latter shows one missense mutation among 83 MPM specimens (1.2%). On the other hand, MPM cells over-express *PDGFRB* mRNA [17] and 20-40% of MPM specimens show immuno-reactivity for PDGFR beta-chain. Normal mesothelium usually does not show PDGFRB mRNA and protein expression [18]. Thus, it should be hypothesized that the tumor progression in MPM is driven by PDGFRB over-expression rather than its activating mutations. This suggests a completely different mechanism to that observed in GIST, where somatic mutations are frequently found within PDGFRA. A generalized overexpression of PDGFRB could be explained by a gain of the copy number of this gene. Preliminary observations at low resolution using fluorescence in situ hybridization suggested that less than 10% of MPM patients could show this phenomenon [19]. However, a search in TGCA portal showed that none of the 83 specimens had significant chromosomal gain/losses of the locus. Overall, these results suggest that the increased expression of PDGFRB should be ascribed to changes at transcriptional level and not to gene amplification.

It was also suggested that the cell growth stimulation triggered by PDGFRB could be caused by an autocrine loop, leading to the activation of tyrosine kinase receptors in MPM cells. The finding that ligands activating PDGFR populate the tumor stromal microenvironment seems to corroborate this hypothesis [20]. Thus, overall, these data suggest that PDGFRB is an important receptor involved in MPM progression. However, more research is warranted in order to provide further evidences. Thus, we analyzed the impact of PDGFRB gene silencing on MPM cell proliferation, cell cycle, apoptosis induction, and invasive behavior on a panel of cell lines, in order to widen the cancer representativeness. The transient PDGFRB-silencing caused a decrease in the proliferation rate and a reduced colony formation capacity of Mero-14 and IstMes2 cell lines, as well as in anchorage-independent growth conditions. Moreover, PDGFRB depletion was associated with an increase in the share of cells in sub-G_1_ phase, as well as with an increased apoptotic rate of these cells. These data are in agreement with those observed by McGary, who demonstrated that the selective inhibition of the PDGFR tyrosine kinase through STI571 slowed PDGF-mediated growth and led to apoptosis of osteosarcoma cells *in vitro* [8]. In addition, Wang and co-workers showed that PDGFRB inhibition alone reduces spontaneous growth and metastasis in Ewing Sarcoma (EWS) [21]. In further work they demonstrated that PDGFRB depletion is fundamental in increasing the activity of TRAIL (tumor necrosis factor-related apoptosis-inducing ligand) for the significant reduction of growth of EWS, as well as decreasing the number of incidences of spontaneous EWS pulmonary metastasis [22]. In a previous work, Zhang and co-workers showed that altered phosphorylation at the tyrosine kinase domain may potentially induce impaired signaling via the PDGF-B/PDGFRβ pathway, triggering a series of intracellular kinase cascades, such as those of Ras/Raf, MAPK, PI3K/AKT and FAK [23]. Their activation would then act as a proliferative stimuli and lead to the inhibition of apoptosis [24–26].

IstMes2 and Mero-14 cells underwent an accumulation in G_2_/M phase after siPDGFRB treatment. This data suggests that inhibition of *PDGFRB* could determine the G_2_ checkpoint recovery arrest. Indeed, a role of PDGFRB in the G_2_ checkpoint was suggested in work by Sun and co-workers [27]. More recently, in support of this hypothesis, it has been shown that imatinib delayed recovery from checkpoint arrest and inhibited the subsequent S-G_2_-M transition, through a persistent activation of ATR-Chk1 signaling, known to be fundamental for the maintenance of G_2_ checkpoint arrest. For this reason, the authors suggested that imatinib may inhibit resumption of tumor proliferation after chemotherapy [28].

Loss of migration ability in the IstMes2 cell line, following *PDGFRB* siRNA administration, was also observed. This data is in agreement with studies highlighting how PDGFRB activity is involved not only in cell growth, but also in the MPM chemotaxis. To support these results, experiments using UTI, a urinary trypsin inhibitor that neutralizes the activation of PDGFRβ cascade, showed that migration of MPM cells is inhibited [29]. Moreover, Abouantoun and co-workers showed PDGFRB tyrosine kinase activity is critical for migration and invasion of medulloblastoma cells, possibly by trans-activating EGFR, and thus its depletion may represent an important therapeutic strategy for the treatment of this cancer [30].

Therefore, our results have shown that reverting *PDGFRB* over-expression can affect cell growth, migration and apoptosis in MPM cell lines. Interestingly, non-malignant cells (Met5A) did not seem to be affected by the *PDGFRB* silencing, suggesting that this gene might be an effective therapeutic target. These evidences together with the fact that a plethora of PDGFRB inhibitors are currently employed in a series of clinical trials, prompted us to investigate whether the phenotypic effect obtained with transient transfection of MPM cell lines could be confirmed through the use of drug inhibitors such as crenolanib or imatinib.

The similar results observed between crenolanib and siPDGFRB confirmed that the effects induced by this drug could be largely mediated through PDGFRB inactivation (being crenolanib designed to be a specific PDGFR inhibitor). Interestingly, both crenolanib and imatinib were effective on Mero-25 cells, that did not respond well to gene silencing, likely due to the weak transfection efficiency found with this cell line. Alternatively, crenolanib and imatinib could inhibit other tyrosine kinase receptors activities inducing increased effects on -Mero-25 cells. In fact, we are aware that the specificity of these inhibitors on PDGFRB is reduced as compared to that of gene silencing. This may explain the variable responses observed among the different MPM cell lines, also in relation to the different endogenous levels of PDGFRA and FLT3 expressed by Mero-14, IstMes2, and Mero-25 cells (Supplementary Figure 1). Indeed, crenolanib is a potent and selective inhibitor also of PDGFRA and FLT3 (the FMS-related tyrosine kinase 3), whereas imatinib is a tyrosine kinase inhibitor (TKI) targeting also BCR-ABL, KIT, and PDGFRA.

As regard imatinib, Bertino and co-workers [2], have reported that this drug has the ability to induce cytotoxicity and apoptosis selectively on PDGFRB positive MPM cells via blockade of receptor phosphorylation and interference with the Akt pathway. To our knowledge, crenolanib has never been tested in MPM. We compared the effect of both the inhibitors, clearly establishing the major efficacy of crenolanib on blocking the malignant phenotype of MPM cells. Indeed, crenolanib has been shown as more efficient than imatinb, even at lower doses, with a Ki about 30 times less than imatinib. Crenolanib is a potent class III receptor tyrosine kinase inhibitor including PDGFRB, with minimal toxicity, whose therapeutic efficacy has already been validated through clinical trials in GIST and human acute myeloid leukemia. Moreover, preclinical evidences showed benefit for lung cancer patients with deregulated PDGFR signaling [31].

Thus, our findings provided a rationale to explore further the efficacy of crenolanib in MPM patients, when characterized by an over-expression of PDGFRB, with promising results greater than those obtained with the use of imatinib. Indeed, previous clinical trials suggested that imatinib, as a single agent, has a limited efficacy in MPM patients [32,33]. The introduction of crenolanib may represent a robust strategy to improve the survival of MPM patients who present with PDGFRB positivity.

In conclusion, this study highlights the importance of the role played by PDGFRB in the MPM malignancy and has provided a rationale for its use as a potential therapeutic target. We also suggest that crenolanib could be an important therapeutic option for MPM patients, which warrants further investigation in order to explore its usefulness as a form of personalized therapy for this cancer type.

## MATERIALS AND METHODS

### Tissue collection and DNA sequencing

Ninety-six samples from formalin fixed and paraffin embedded tissues (FFPE) of MPM patients hospitalized and surgically resected for the tumor were enrolled for *PDGFRB* mutation screening. According to the Helsinki declaration, the local ethical committee approved the study. All tumor samples used in this study were selected and dissected by an experienced pathologist. Demographic information is reported in Table S3. FFPE sections were de-paraffined by submersion in xylene; the tissue was then incubated overnight at 56°C with Proteinase K (Qiagen, UK) to allow samples lysis and DNA was extracted using the Trizol reagent (Invitrogen, CA, USA) following the manufacturer’s instructions. To obtain RNA free genomic DNA, an RNase A (Qiagen, UK) treatment was performed following the protocol instructions. The concentration and purity of the isolated DNA were measured using a NanoDrop ND-1000 Spectrophotometer (Thermo Fisher Scientific, DE, USA). Subsequently, the mutation screening was performed with automatic sequencing (Sanger reaction), according to the standard protocol. Specifically, the whole exons 12 and 18 of *PDGFRB* were PCR-amplified from genomic DNA using the specific primer pairs: Forward= tgtcctagacggacgaacct (Exon 12) and gaagggtctttccccacaat (Exon 18) Reverse= ccaacttgagtccccacact (Exon 12), and cacactggtcaggagggaat (Exon 18) and sequenced using PCR oligonucleotide as sequencing primer.

Copy number alterations data of 83 MPM patients were obtained from Cancer Genome Atlas (TCGA) consortium.

### Cell cultures

The mesothelial non-MPM immortalized cell line (Met5A), was purchased from ATCC and grown in Medium 199 with HEPES (Life Technologies, Monza, Italy) supplemented with 10% FCS, 3.3 nM epidermal growth factor (EGF, Life Technologies, Monza, Italy), 400 nM hydrocortisone (Sigma Aldrich Corp. St Louis, MO, USA), and 870 nM insulin (Life Technologies, Monza, Italy). Three mesothelioma cell lines (Mero-14, Mero-25, and IstMes2), kindly donated by Istituto Tumori of Genova (Italy), were cultured in DMEM medium (Lonza, Basel, Switzerland), with 10% of FCS.

### PDGFRB inhibitors and PDGFRB silencing RNA oligonucleotide

Imatinib (Cayman Chemical, Michigan, USA) and Crenolanib (Selleckman) were dissolved in DMSO to give a final concentration of 10 mM, and used at the previously established IC_30_ concentration, that is of 25 μM and 1 μM respectively. The following primary antibodies were used: primary mouse monoclonal anti-PDGFRB, anti-PDGFRA, and anti-FLT3 (Santa Cruz, TX, USA; 1:300) or mouse monoclonal anti-ß-actin (Anti-Actin, Clone C4, Millipore, MA, USA; 1:5000). IgG-HRP Santa Cruz (1:5000) was used as secondary antibody. Different silencing-RNAs (siRNAs) were tested, and purchased from Qiagen (Qiagen, UK). Further experiments were performed by using a single specific siRNA for *PDGFRB* (SI00605738, the so-called “siPDGFRB” now-on) and the “AllStars Negative Control siRNA” (SI03650318, used as non-targeting control - the so-called “C-PDGFRB” now-on). siRNA oligonucleotides were re-suspended in the provided buffer at a final stock concentration of 20 mM, and employed at 50nM in each experiments. HiPerfect transfection reagent was employed for siRNA trasfection (Qiagen, UK), as previously described [16].

### RNA isolation, Quantitative Real-Time PCR (RT-qPCR) and Western Blotting

These three techniques were performed as previously described [16,34]. Densitometry results of western blot were analysed with Image J software (NIH, Bethesda, MD, USA). For quantitative analysis, the signal intensity of each band was normalized with ß-actin densitometry values. Each density was compared to the intensity of the reference cell line (Met5A) and all western blot analyses were replicated twice for the calculation of the average increase.

### Sulphorhodamine (SRB) assay

Cells were seeded in 96-well plates at a density of 3 × 10^3^. The next day, (day 0), one plate was assessed. The remaining plates were tested at 2-day intervals for a total of 8 days. Cells were fixed with 100 μL per well of ice-cold 40% (vol/vol) TCA (Sigma Aldrich Corp. St Louis, MO, USA), and then stained with 0.4% SRB solution (Sigma Aldrich Corp. St Louis, MO, USA). After staining, unbound dye was removed by washing five times with 1% acetic acid solution and left to air dry. The bound SRB dye was then solubilized by adding unbuffered Tris-base solution, and plates were then read at OD 492 nm, using a microplate reader.

### Determination of drugs’ doses

Sub-confluent cells in 96-well plates were exposed for 48 h to medium supplemented with 2% FBS, with or without crenolanib (in a range of concentrations from 20 μM to 0.3 μM) or imatinib (in the range from 200 μM to 0.1 μM). The different concentration ranges have been chosen based on previous literature searches. Cell viability was assessed by SRB assay on 3 replicates at each concentration point to determine single drug lethal concentration values and their critical IC_50_ and IC_30_ values. Normalised cytotoxicity percentages were obtained from the formula: (A_570_ mean values of extracts from treated samples/A_570_ mean values of extracts from untreated control samples) × 100. Because the dose-response relationship was similar in all employed cell lines (data not shown for brevity), the unique dose of 1μM for crenolanib and 25μM for imatinib (corresponding to the IC_30_ for both of them) was used for all the assays.

### Colony formation assays

3×10^3^ cells were seeded in a 96-well plate and, after 24 h, transfected with the correct concentration of siPDGFRB, or treated with PDGFRB inhibitors. After 24 h post treatment, cells were transferred in 6-well plates and incubated for a further 14 days. Following 14 days incubation, growth medium was removed and cells fixed and stained in 10% ethanol solution containing 0.1% crystal violet for 1 h. Colonies were then counted and measured with Image J software.

### Flow cytometry (FACS)

After siRNA transfection or drug treatments for 72 h, 10^5^ cells were collected, washed in phosphate-buffered saline (PBS), pelleted by centrifugation and fixed in 70% ethanol. Immediately prior to staining, cells were suspended in PBS containing 50 μg/ml of RNAse A (Qiagen, UK), and then, stained with propidium iodide (final concentration 100 μg/ml) overnight at 4°C. The percentage of cells in subG_1_, G_0_/G_1_, S and G_2_/M phases were determined from 10.000 cells using the BD C6 ACCURI Software (Becton Dickinson). The experiments were carried out three independent times (triplicates).

### Caspase - GloH 3/7 assay

Caspase-3/7 activation was measured using the Caspase-Glo 3/7 Luminescence Assay (Promega Corp. Madison, Wisc., USA) according to the manufacturer’s instructions. In a 6-well plate 3×10^5^ cells were incubated. The day after, the cells were treated with siRNAs or PDGFRB inhibitors, for 72 h. Then, the cells were collected by trypsinization, and approximately 15×10^3^ cells were transferred in a 96-well white plate. Caspase-3/7-Glo reagent was added, and the samples were incubated at 37°C for 1 h. The luminescence that is proportional to the caspase 3/7 activities was determined by luminometer (Tecan Sunrise, Austria GMBH).

### Wound-Healing Assay

25×10^3^ cells were seeded in a 6-well plate and, after 24h, transfected with siRNAs or PDGFRB inhibitors for 72 h. A linear scratch in the confluent cell monolayer was made with a sterile pipette tip after 39 h (time optimized following preliminary trials) following siRNA transfection. Then, cells were rinsed and incubated in full medium. Finally, cells were photographed at 72 h (i.e. 33h following the scratch) with an optical microscope at 10X magnification connected to a computer. The migration was then evaluated on the images, and measured using Image J software.

### Soft agar assay for colony formation

Base layer of soft agar was prepared by pouring 1 mL of 0.9% agar in MPM cells media in each well of 6-well plates and allowed to polymerize for few minutes. Top layer of soft agar was prepared using 0.45% agar in MPM cells media and cells from each treatment groups (C-PDGFRB vs siPDGFRB; DMSO vs crenolanib and imatinib) were mixed (10.000 cells in 1 mL soft agar solution) and plated over the base layer in each well. All the soft agar plates were allowed to solidify and then maintained at 37°C with a humidified atmosphere containing 5% CO2. Colony formation of cells on soft agar was monitored by microscopic observation on daily basis. On Day 14, colonies were counted manually using microscope and representative images were taken.

### Statistical analyses

The measurements of gene expression performed on cell lines, and the results obtained from the *in vitro* assays were statistically evaluated using a two-tailed Student’s t-test. The effects of the combination of treatments (drugs +/− siRNAs) were evaluated with a multifactor analysis of variance (MANOVA) model. The statistics were performed with the software Statgraphics Centurion XV (StatPoint, Inc.).

## SUPPLEMENTARY MATERIAL FIGURE AND TABLES


