# Supplementary material for: Inhibition of the platelet-derived growth factor receptor beta (PDGFRB) using gene silencing, crenolanib besylate, or imatinib mesylate hampers the malignant phenotype of mesothelioma cell lines
Source: Genes Cancer. 2017 Jan;8(1-2):438–52. doi: 10.18632/genesandcancer.129 (PMC5396622; doi:10.18632/genesandcancer.129)
Supplement: Supplementary file 1 [file ganc-08-438-s001.pdf]

Inhibition of the platelet-derived growth factor receptor beta (PDGFRB) using gene silencing, crenolanib besylate, or imatinib mesylate hampers the malignant phenotype of mesothelioma cell lines – Melaiu et al

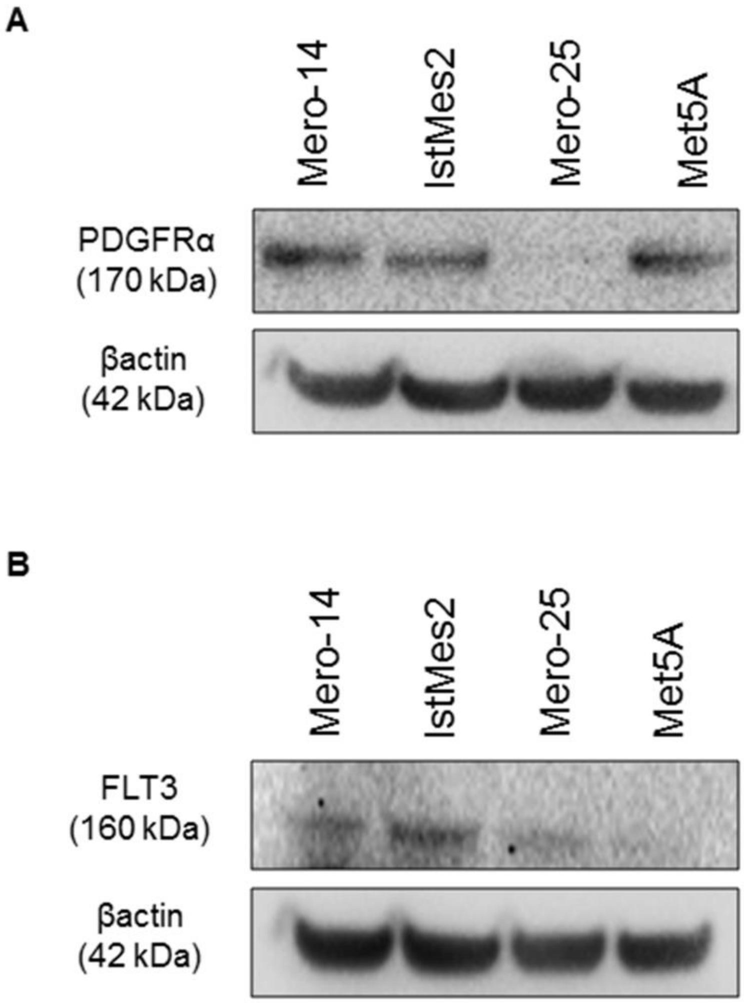

Supplementary Figure 1: A. Protein levels of PDGFRA in Mero-14, IstMes2, and Mero-25 human MPM cell lines and Met5A. **B**. Protein levels of FLT3 in Mero-14, IstMes2, Mero-25, and Met5A cells. β-actin was used as reference. The protein levels were confirmed by two independent experiments.

Table S1: Results of the mutation screening of *PDGFRB* (wt: wild type; NA: no-amplification).

Number of MPM	Exon 12	Exon 18
88	wt	wt
4	NA	NA
2	NA	wt
1	wt	NA
1	wt	c.2523G>A p.K841K

Table S2: Tables (A-C) showing the breakdown of the Flow data (G_0/G_1, S, G_2/M, sub-G_1,) for the treatments with siPDGRB, abbreviated as siPDG. (A), Crenolanib, abbreviated as CREN. (B). and Imatinib abbreviated as IMAT (C). Means and standard error of the mean (SEM) are reported for each treatment derived from 3 independent experiments.

**A**	**MERO-14**				**MERO-25**				**ISTMES2**				**MET5A**			
	C-	SEM	siPDG	SEM	C-	SEM	siPDG	SEM	C-	SEM	siPDG	SEM	C-	SEM	siPDG	SEM
**G_0/G_1**	75,58	0,01	67,74	0,05	65,45	0,02	62,5	0,00	61,22	0,04	45,63	0,01	34,66	0,02	33,0	0,01
**S**	6,37	0,03	8,85	0,01	6,88	0,04	8,04	0,03	13,64	0,01	13,17	0,00	6,04	0,03	7,13	0,01
**G_2/M**	11,28	0,02	12,86	0,01	23,99	0,02	25,01	0,04	20,45	0,03	34,09	0,02	57,74	0,04	58,6	0,00
**SubG_1**	6,77	0,01	10,55	0,06	3,68	0,00	4,45	0,00	5,05	0,02	7,11	0,01	1,56	0,02	1,24	0,03

**B**	**MERO-14**				**MERO-25**				**ISTMES2**				**MET5A**			
	DMSO	SEM	CREN	SEM	DMSO	SEM	CREN	SEM	DMSO	SEM	CREN	SEM	DMSO	SEM	CREN	SEM
**G_0/G_1**	79,96	0,03	65,53	0,02	69,81	0,01	51,70	0,06	73,01	0,07	62,88	0,04	34,78	0,02	37,77	0,02
**S**	5,24	0,01	5,65	0,03	7,51	0,01	9,09	0,03	9,47	0,05	5,87	0,00	6,28	0,04	5,29	0,05
**G_2/M**	7,70	0,03	3,74	0,01	20,09	0,04	32,81	0,05	12,74	0,00	21,41	0,05	57,80	0,04	55,45	0,03
**SubG_1**	7,1	0,02	25,08	0,05	2,59	0,01	6,4	0,00	4,78	0,02	9,84	0,01	1,14	0,01	1,49	0,00

**C**	**MERO-14**				**MERO-25**				**ISTMES2**				**MET5A**			
	DMSO	SEM	IMAT	SEM	DMSO	SEM	IMAT	SEM	DMSO	SEM	IMAT	SEM	DMSO	SEM	IMAT	SEM
**G_0/G_1**	79,96	0,03	76,32	0,03	69,81	0,01	70,45	0,04	73,01	0,07	62,74	0,00	34,78	0,02	31,72	0,03
**S**	5,24	0,01	5,49	0,02	7,51	0,01	6,99	0,01	9,47	0,05	9,52	0,01	6,28	0,04	9,61	0,01
**G_2/M**	7,70	0,03	6,17	0,03	20,09	0,04	15,66	0,02	12,74	0,00	19,43	0,02	57,80	0,04	54,54	0,01
**SubG_1**	7,1	0,02	12,02	0,03	2,59	0,01	6,9	0,01	4,78	0,02	8,31	0,01	1,14	0,01	4,13	0,04

Table S3. Characteristics of volunteers enrolled into the study.

ID	Sex	Year of birth	Histotype	ID	Sex	Year of birth	Histotype
M1	M	1940	Sarcomathoid	M49	F	1947	Epithelioid
M2	F	1935	Epithelioid	M50	M	1936	Bifasic
M3	M	1951	Epithelioid	M51	M	1932	Epithelioid
M4	M	1944	Epithelioid	M52	M	Unknown	Epithelioid
M5	F	1935	Epithelioid	M53	M	1944	Epithelioid
M6	M	1957	Bifasic	M54	F	1932	Bifasic
M7	F	1933	Epithelioid	M55	M	1936	Epithelioid
M8	F	1950	Epithelioid	M56	M	1943	Epithelioid
M9	M	1941	Bifasic	M57	M	1939	Epithelioid
M10	F	1936	Epithelioid	M58	M	1934	mesenchimorph
M11	F	1973	Epithelioid	M59	M	1938	Epithelioid
M12	M	1949	Epithelioid	M60	M	1937	Sarcomathoid
M13	M	1954	Desmoplastic	M61	M	1941	Epithelioid
M14	F	1953	Epithelioid	M62	M	1951	Epithelioid
M15	F	1940	Epithelioid	M63	M	1937	Epithelioid
M16	M	2008	Epithelioid	M64	M	1933	Epithelioid
M17	M	1938	Epithelioid	M65	M	2009	Desmoplastic
M18	M	1945	Epithelioid	M66	M	1947	Epithelioid
M19	M	1938	Epithelioid	M67	M	1932	Epithelioid
M20	M	1947	Sarcomathoid	M68	M	1934	Epithelioid
M21	M	1931	Sarcomathoid	M69	F	1952	Anaplastic
M22	M	1937	Sarcomathoid	M70	M	1930	Epithelioid
M23	M	1946	Epithelioid	M71	M	1936	Epithelioid
M24	M	1949	Bifasic	M72	M	1934	Bifasic
M25	M	1944	Epithelioid	M73	M	1961	Epithelioid
M26	F	1924	Epithelioid	M74	M	1963	Epithelioid
M27	F	1936	Epithelioid	M75	M	1936	Epithelioid
M28	M	1951	Epithelioid	M76	M	1938	Epithelioid
M29	M	1940	Bifasic	M77	M	1948	Epithelioid
M30	M	1933	Epithelioid	M78	M	1928	Bifasic
M31	M	1932	Epithelioid	M79	M	1945	Epithelioid
M32	M	1937	Epithelioid	M80	F	1933	Epithelioid
M33	M	1941	Epithelioid	M81	M	1941	Epithelioid
M34	M	1939	Epithelioid	M82	M	1940	Epithelioid
M35	M	1941	Epithelioid	M83	M	1952	Epithelioid
M36	M	1939	Epithelioid	M84	M	1949	Epithelioid
M37	M	1941	Epithelioid	M85	M	1951	Epithelioid
M38	M	1934	Epithelioid	M86	M	1932	Epithelioid
M39	M	1931	Bifasic	M87	M	1938	Bifasic
M40	M	1937	Sarcomathoid	M88	M	1937	Epithelioid
M41	M	1938	Sarcomathoid	M89	M	1936	Epithelioid
M42	M	1941	Epithelioid	M90	M	1933	Bifasic-Epithelioid
M43	M	1932	Epithelioid	M91	M	1954	Epithelioid
M44	M	1938	Epithelioid	M92	M	1939	Sarcomathoid
M45	M	1934	Epithelioid	M93	M	1963	Epithelioid
M46	F	1937	Epithelioid	M94	M	1949	Epithelioid
M47	M	1938	Sarcomathoid	M95	M	1930	Epithelioid
M48	M	1951	Epithelioid	M96	M	1956	Epithelioid
